# Supplementary material for: Polymorphisms in STING Affect Human Innate Immune Responses to Poxviruses
Source: Front Immunol. 2020 Oct 14;11:567348. doi: 10.3389/fimmu.2020.567348 (PMC7591719; doi:10.3389/fimmu.2020.567348)

Supplementary Material

Polymorphisms in STING affect human innate immune responses to poxviruses

**Richard B. Kennedy, PhD^1*^; Iana H. Haralambieva, PhD^1^; Inna G. Ovsyannikova, PhD^1^; Emily A. Voigt, PhD^1^; Beth R. Larrabee^2^; Daniel J. Schaid, PhD^2^; Michael T. Zimmermann, PhD^3^; Ann L. Oberg, PhD^2^; Gregory A. Poland, MD^1^**

^1^Mayo Clinic Vaccine Research Group, Mayo Clinic, 200 First Street SW, Rochester, MN, 55905 USA

^2^Division of Biomedical Statistics and Informatics, Department of Health Science Research,

Mayo Clinic, 200 First Street SW, Rochester, MN 55905 USA

^3^Bioinformatics Research and Development Laboratory, Genomics Sciences and Precision Medicine Center, Medical College of Wisconsin, 8701 Watertown Plank Rd., Milwaukee, WI, 53226, USA

**
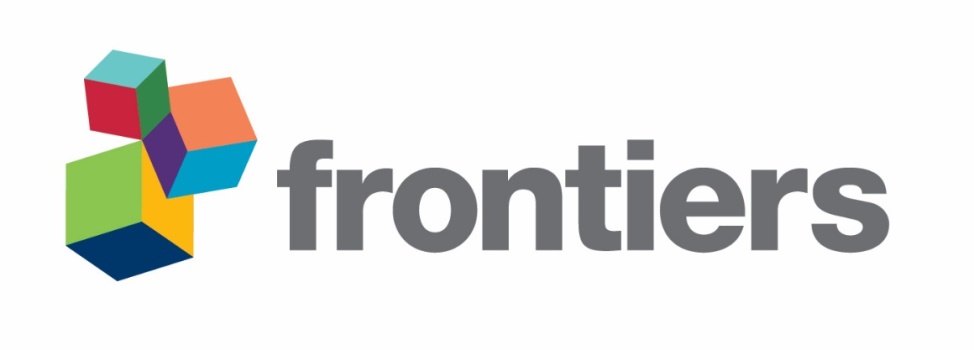
**

# Supplementary Figures and Tables

**Supplementary Table 1**

| **Supplementary Table 1. Cohort Demographics** | |
| --- | --- |
| *Total subjects* | 1,653 |
| *Gender* |  |
| Male | 1,365 (82.6%) |
| Female | 288 (17.4%) |
| *Age* | 19 – 41 years old |
| *Ethnicity* |  |
| Hispanic/Latino | 381 (23%) |
| Not Hispanic/Latino | 1248 (75.5%) |
| Unknown | 24 (1.5%) |
| *Race* |  |
| American Indian/Alaska Native | 26 (1.6%) |
| Asian, Native Hawaiian, Pacific Islander | 12 (0.7%) |
| African American | 25 (1.5%) |
| Caucasian | 1,351 (81.7%) |
| More than one race | 81 (4.9%) |
| Other | 120 (7.3%) |
| Unknown | 38 (2.3%) |
| *Time since immunization* |  |
| Median | 1.7 years |
| Range | 1.1 – 2.8 years |

**Supplementary Figure 1.**


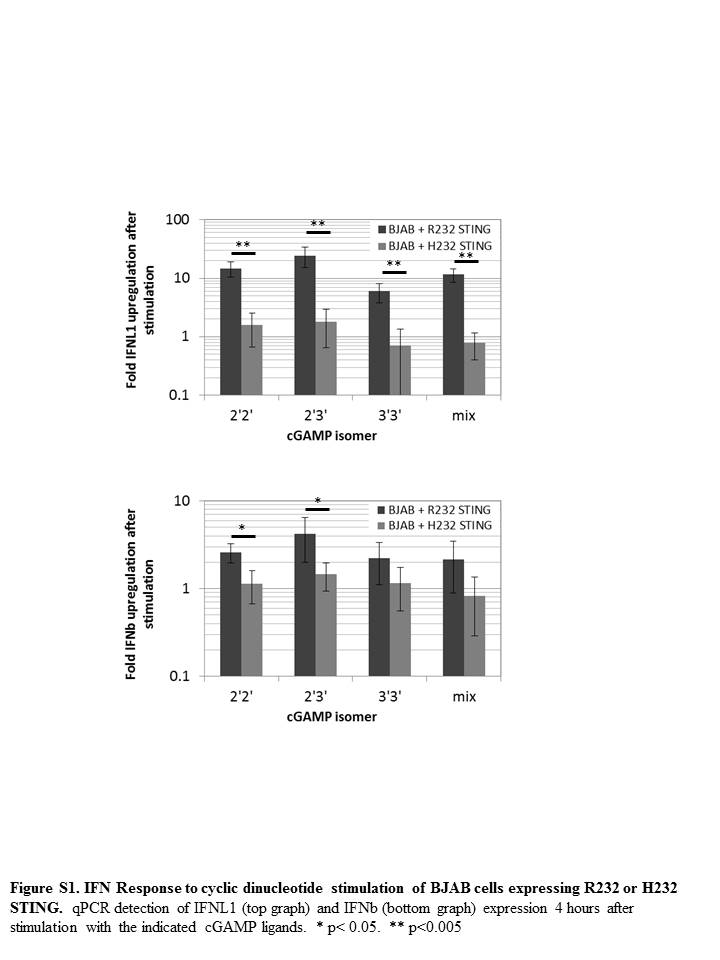


**Supplementary Figure 2.**


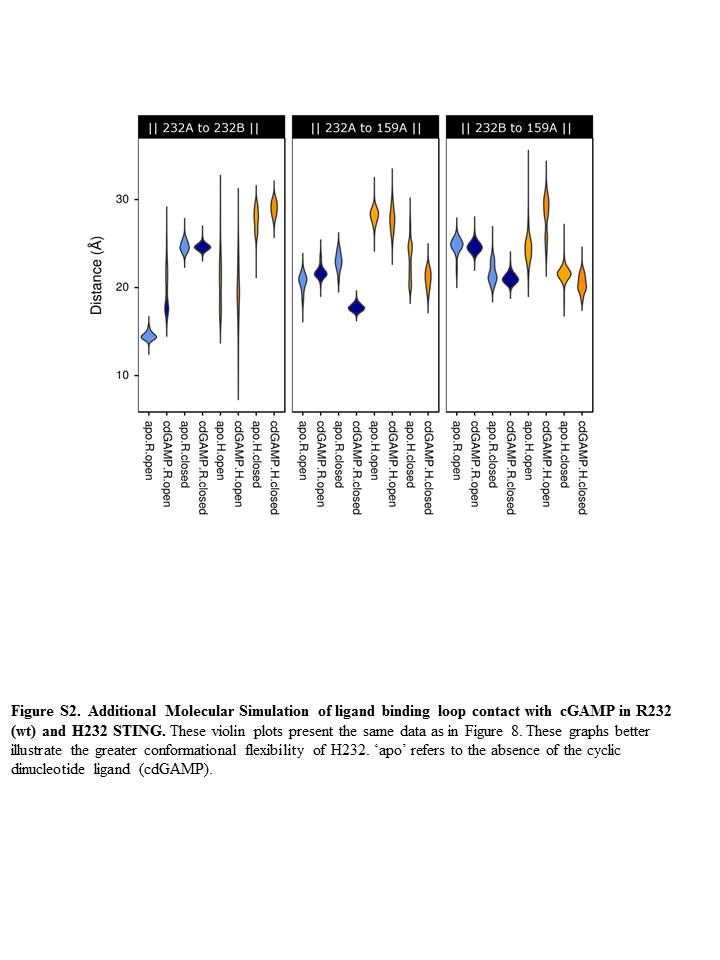

Supplement: Supplementary file 1 [file Table_1.docx]
